# Insomnia among patients with advanced disease during admission in a Palliative Care Unit: a prospective observational study on its frequency and association with psychological, physical and environmental factors

**DOI:** 10.1186/1472-684X-13-40

**Published:** 2014-08-12

**Authors:** Anna Renom-Guiteras, José Planas, Cristina Farriols, Sergi Mojal, Ramón Miralles, Maria A Silvent, Ada I Ruiz-Ripoll

**Affiliations:** 1 School of Nursing Science and Institute of General Practice and Family Medicine, Faculty of Health, University of Witten/Herdecke, 50 Alfred-Herrhausen-Str, 58448 Witten, Germany; 2 Geriatric Service, Hospital de l’Esperança, Centre Fòrum, Hospital del Mar, Parc de Salut Mar, Barcelona, Spain; 3 Palliative Care Unit, Department of Medical Oncology, Hospital de l’Esperança, Parc de Salut Mar, Barcelona, Spain; 4 Universitat Autònoma de Barcelona, Barcelona, Spain; 5 IMIM (Hospital del Mar Medical Research Institute), Barcelona, Spain; 6 Scientific and Technical Services, IMIM (Hospital del Mar Medical Research Institute), Barcelona, Spain; 7 Department of Psychiatry, Institute of Neuropsychiatry and Addiction, Hospital del Mar, Parc de Salut Mar, Barcelona, Spain

**Keywords:** Palliative care [MeSH], Sleep disorders [MeSH], Insomnia, Rumination, Oncology, Symptom assessment [MeSH], Sleep Disturbance Scale

## Abstract

**Background:**

The aims of this study were: 1) to assess the frequency of insomnia among patients during admission in a Palliative Care Unit (PCU); 2) to study the association between emotional distress and insomnia, taking physical, environmental and other psychological factors into account.

**Methods:**

Prospective observational study including patients consecutively admitted to a PCU during eight months, excluding those with severe cognitive problems or too low performance status. Insomnia was assessed by asking a single question and by using the Sleep Disturbance Scale (SDS), and emotional distress using the Hospital Anxiety and Depression Scale (HADS). Physical, environmental and other psychological factors potentially interfering with sleep quality were evaluated. Association between insomnia and the factors evaluated was studied using univariate and multivariate regression analyses.

**Results:**

61 patients were included (mean age 71.5 years; 95% with oncological disease); 38 (62%) answered “yes” to the insomnia single question and 29 (47%) showed moderate to severe insomnia according to the SDS. 65% showed clinically significant emotional distress and 79% had nocturnal rumination. The physical symptoms most often mentioned as interfering with sleep quality were pain (69%) and dyspnoea (36%). 77% reported at least one environmental disturbance. In the univariate analysis, answering “yes” to the insomnia single question was significantly associated with higher HADS score, anxiety, nocturnal rumination, clear knowledge of the diagnosis, higher performance status and dyspnoea; moderate to severe insomnia was significantly associated with nocturnal rumination, higher performance status, environmental disturbances and daytime sleepiness. In the multivariate regression analysis, answering “yes” to the single question was associated with dyspnoea (OR 7.2 [1.65-31.27]; p = 0.009), nocturnal rumination (OR 5.5 [1.05-28.49]; p = 0.04) and higher performance status (OR 14.3 [1.62-125.43]; p = 0.017), and moderate to severe insomnia with nocturnal rumination (OR 5.6 [1.1-29.1]; p = 0.041), and inversely associated with daytime sleepiness (OR 0.25 [0.07-0.9]; p = 0.043).

**Conclusions:**

Insomnia was highly frequent. Several physical, psychological and environmental factors seemed to influence insomnia. Within the multimodal management of insomnia, the assessment of nocturnal rumination may be of particular interest, irrespective of emotional distress. Further studies with larger sample sizes could confirm this result.

## Background

The prevalence of insomnia among persons with cancer is higher than in the general population (up to 50% versus 4-22%) [1-3]; its prevalence among patients with advanced cancer admitted to Palliative Care Units (PCUs) has been estimated as being between 45% and 95% [4,5]. Nevertheless, insomnia has received little attention from the oncology community compared with other symptoms such as pain and fatigue [6].

Insomnia is defined as the subjective perception of difficulty with sleep initiation, duration, consolidation, or quality that occurs despite adequate opportunity for sleep and results in some form of daytime impairment [7]. Insomnia is strongly correlated with patient satisfaction and with quality of life in cancer [6,8]. It brings patients discomfort and suffering, and has physical and psychological consequences such as increase of fatigue, pain intolerance, irritability and depressive mood [6,9]. However, patients tend to underreport insomnia [10], and the routine clinical assessment of insomnia is often suboptimal.

The primary goal of the treatment of insomnia should be first to relieve any underlying disorder that may be causing the sleep disturbance. As a variety of factors may influence insomnia in hospitalised patients suffering from advanced cancer, a multimodal treatment, including both pharmacologic and non-pharmacologic therapies, should be considered. A plan combining attention to sleep hygiene and cognitive-behavioral therapy with prescription of hypnotic medications may help to relieve insomnia in cancer patients and improve their quality of life [6].

In the literature, a variety of factors that may influence sleep quality among palliative care patients have been mentioned. The factors associated with insomnia most commonly cited are pain and psychological symptoms. Uncontrolled pain, pain treatment and “interference of pain with mood” have been suggested as being associated with insomnia [11-13], as well as low or variable moods, dreams, concerns, hopelessness, post-traumatic experience, anxiety and depression [4,11,13-16]. Anxiety has been associated with difficulties in falling asleep, with less restoring sleep and with nightmares, while depression has been associated with early awaking, non-restorative sleep, fatigue, nightmares and insomnia according to the Pittsburgh Sleep Quality Index [5,15]. However, only a few studies have used specific and validated tools for the assessment of psychological symptoms in relation to insomnia [5,11].

Other factors which could influence insomnia in cancer population are age, performance status and certain pharmacological treatments [11,14]. Female gender and non-white race, as well as excessive consumption of coffee and/or alcohol, or certain physical problems like chronic kidney disease may also increase the risk of incident insomnia in the general population [17]. Empirical evidence suggests that biological factors such as cytokines [18,19], genetic and metabolic disorders [20] and cortisol awaking response [19,21] can contribute to the disruption of the sleep cycle.

Environmental factors, and especially those in PCUs as for example interruptions during the night by the nursing staff, noise coming from the ward or from another patient in the same room, or even having a room for individual use, could influence the sleep quality of the patients during admission. However, most studies evaluating the factors associated with insomnia have not taken environmental factors into account.

Patients are admitted to PCUs for rapid symptom control and intensive psychosocial care, normally for relatively short periods. The length of stay may vary from two days to more than two weeks [22,23]. Disturbing symptoms are evaluated daily by professionals in PCUs, as their intensity and characteristics can change from one day to the next. Evaluating the presence of insomnia specifically during admission to PCU, instead of evaluating persistent or chronic insomnia, may help professionals to adapt the management of insomnia, taking the changing situation of symptoms, possible acute disease and type of unit into account. Studies evaluating the prevalence of insomnia in this population have often used instruments which refer to the preceding 15 days or four weeks or which take relatively long to respond to, making their use more difficult and less specific for the evaluation of insomnia during admission in PCUs [24]. Other studies have used a single question [12,25].

The aims of the present study were: 1) to evaluate the frequency of insomnia during hospitalisation among the patients admitted to a PCU by asking a single question and by using an assessment tool that fits the characteristics of this population (the 3-item questionnaire Sleep Disturbance Scale) and 2) to study the association between insomnia and emotional distress, considering both anxiety and depression and using a validated tool, and taking also physical, environmental and other psychological factors into account.

## Methods

### Study design

A prospective observational study design was used.

### Participants and setting

Participants were patients admitted consecutively to the PCU in a university hospital in Barcelona, Spain. This PCU receives patients referred from both acute care hospitalisation units and home care and has a mean length of stay of 15 days. A sample size of 60 patients was calculated to detect a difference of 35% in the presence of clinically significant emotional distress between patients with and without insomnia, considering that 50% of the patients would suffer from insomnia and assuming an alpha error of 5% and statistical power of 80%. Data inclusion went on over an eight-month period and finished in October 2009. The inclusion criteria were: 1) being admitted to PCU during this period; 2) acceptable performance status (defined as Karnofsky Index ≥ 30); 3) acceptable cognitive status (defined as Pfeiffer Index ≤ 4 errors); and hospital stay for > 2 nights.

### Procedure

A Case Report Form (CRF) was developed after a literature review. Approval of the study protocol and the CRF was obtained by the hospital’s Ethical Committee for Clinical Research (*Comitè Ètic d’Investigació Clínica del Parc de Salut Mar*). The study was conducted in accordance with the Declaration of Helsinki. The interviewers assessed the newly admitted patients for eligibility and obtained their oral informed consent for participation. Then the face-to-face interview took place. Finally, a researcher (ARG) extracted demographic data from the computerized database of the hospital.

Interviewers were nursing staff employed at the PCU, and had been trained by a researcher (ARG) or one experienced nurse (MS) to administer the survey in a standardized way.

Interviews were intended to take place between days 3 and 7 of admission. This was achieved for 41 of the participants (67.2%). The interview with 3 participants (4.9%) took place before day 3 and after day 7 with 17 participants (27.9%).

### Measures

#### ***Assessment of insomnia***

Two instruments were considered of interest and applied to all included patients for the assessment of insomnia:

1. *Insomnia single question.* Patients were asked “Have you been suffering from insomnia since you have been admitted?” Possible answers were “yes”, “no” or “occasionally”*.* Although this is a non-validated question, we considered that it could be of clinical interest. A similar question was used in a previous study, in which patients were asked “Do you have trouble sleeping at night?” and given three possible answers (“yes”, “no”, “occasionally”) [12]; while none of the participants answered “no”, the answer “occasionally” may have identified those patients with less insomnia. Therefore, for the deductive analyses of the present study, researchers compared those participants answering “yes” with those answering “no” or “occasionally”.

2. *Sleep questionnaire.* The Sleep Disturbance Scale (SDS), developed in the context of the study by Anderson et al. [26], was chosen because it is short and refers to the previous 24 hours. The SDS assesses three main sleep items: difficulty in falling asleep, waking up during the night and waking up too early in the morning. Patients are asked to score each item on a Likert scale from 0 to 10. An overall sleep disturbance score is calculated as the average of the rated scores of the three sleep items. Patients are classified in those with moderate to severe insomnia (overall sleep disturbance score ≥ 5) and those without moderate to severe insomnia (overall sleep disturbance score < 5). In the original study [26], the SDS demonstrated adequate internal reliability with a coefficient alpha of 0.71. Construct validity was supported by factor analysis and a single underlying construct was identified. The scale includes a final independent item asking patients to judge the total number of hours they sleep per night. Patients are classified in those who report sleeping < 5 hours and those who report sleeping ≥ 5 hours per night. The scale was translated into Catalan by one of the researchers (ARG) and the time reference was widened to “since you have been admitted”.

Additionally, patients were asked to rate how worried about insomnia they were, using a Likert scale from 0 to 5. A score of ≥ 3 was considered as moderate worry. Patients were asked if they suffered from daytime sleepiness, feeling of non-restorative sleep, and when insomnia had started (before diagnosis of the current disease, after diagnosis or after admission to the PCU).

#### ***Assessment of emotional distress and other factors potentially associated with insomnia***

The main outcome variable was emotional distress, which was measured using the Hospital Anxiety and Depression Scale (HADS), a validated 14-item scale which has been used with patients with advanced oncological disease and validated in a Spanish population [27,28]. Clinically significant emotional distress was considered if the HADS score was ≥ 19. HADS includes two subscales, separately evaluating clinical anxiety and depression. A score of ≥ 11 for each subscale indicates anxiety or depressed mood, respectively [29].

Other psychological factors as well as physical and environmental factors potentially associated with insomnia were assessed.

Other psychological aspects evaluated were presence of nightmares, feeling of fear or loneliness and presence of nocturnal rumination, the latter being defined as repetitively thinking about personal difficulties and feelings, their causes, meanings, and consequences [30,31]. The degree of information about the diagnosis and prognosis of the present disease was rated by the interviewer according to his/her impression after the interview.

Physical symptoms were assessed by asking patients whether the following symptoms interfered with their sleep quality: dyspnoea, functional incontinence, fever, sweating, nausea or vomiting, itch, pain, drug or tobacco abstinence, nycturia, cough and restless-legs.

The assessment of environmental factors consisted of asking the patients if light or noise in their shared room or coming from the ward, or interruptions by nurses were interfering with the quality of their sleep. Patients giving a positive answer to any of these questions were considered to have “environmental disturbances”. Furthermore, information was collected as to whether the patient slept in an individual room and whether the family stayed with him/her during the night. Patients were asked if they had sleep habits at home that could not be accomplished at the PCU.

#### ***Patients’ perspective on the group of factors that interfered most***

Finally, patients were asked which of the three mentioned groups of factors interfered most with the quality of their sleep.

#### ***Other variables***

Sociodemographic data, location before admission (home or acute care), death during PCU stay, information on the diagnosis and stage of the disease, comorbidity and medication were collected from the patients’ records in the computerized database of the hospital.

#### ***Data analysis***

All statistical analyses were performed using SPSS 15.0 package (SPSS Inc., Chicago, IL). Means, standard deviations and ranges were calculated for the scale variables. Basic descriptive statistics were computed for the sociodemographic variables, other aspects in relation with insomnia and factors potentially associated with insomnia.

Univariate analyses were performed in order to investigate associations between insomnia and the factors evaluated. Student’s T test, Chi-square and Fisher’s Exact Test were used, depending on the type of the scale and the distribution of the categories. Multivariate logistic regression analyses were performed, including those variables significantly associated with insomnia in the univariate analyses which the researchers judged as clinically meaningful, in order to investigate associations between the factors and presence of insomnia using the two instruments. Manual backward stepwise strategy was used to remove non-significant variables of the model, taking possible confounding factors into account, until a final model was obtained. P values less than 0.05 were considered statistically significant.

## Results

One hundred and seventy-six patients were consecutively admitted to the PCU during the eight-month period. Sixty-one patients (34.7%) fulfilled the inclusion criteria. Thirty-four of them were men and 27 women, and their mean age was 71.5 ± 11 years. The majority of included patients suffered from cancer (95.1%), with a high prevalence of metastatic cancer.

Ninety-five patients (54.0%) could not be included because either they did not give informed consent (n = 2) or they did not fulfil the inclusion criteria because they were too cognitively impaired (n = 34), had a Karnofsky Index < 30 (n = 44), spent less than two nights at the PCU (n = 8), or had a Karnofsky Index < 30 and spent less than two nights at the PCU (n = 7).

Twenty admitted patients (11.4%) were not assessed for inclusion either because they died (n = 5), or were discharged (n = 15) before assessment.

Table 1 shows the characteristics of the patients who were included, those who were excluded and those who were not assessed. Excluded and not assessed patients did not differ significantly from included patients in terms of age, gender, main diagnosis and stage of the disease. However, significantly more patients in the groups of excluded (p < 0.001) and not assessed (p = 0.024) patients died during admission in the PCU than in the group of included patients.

**Table 1:** Sociodemographic and clinical characteristics of the patients

**Variable**	**Included (n = 61)**	**Not included (n = 95)**	**Not assessed (n = 20)**
	n (%)	n (%)	n (%)
Sex (man)	34 (55.7)	54 (56.8)	12 (60.0)
Mean age ± SD, years	71.5 ± 11	73.3 ± 13.23	72.4 ± 10.90
Mean Pfeiffer Index ± SD (range 0-10)^a^	1.3 ± 1.08	not available	not available
Karnofsky Index (range 0-100)^b^			
≤ 50	45 (73.8)	not available	not available
> 50	16 (26.2)	not available	not available
Main diagnosis			
Oncological	58 (95.1)	84 (88.4)	18 (90.0)
Non-oncological	3 (4.9)	11 (11.6)	2 (10.0)
Cancer site			
Lung	13 (21.3)	20 (21.1)	4 (20.0)
Colon	10 (16.4)	7 (7.4)	3 (15.0)
Gynaecological (not breast)	7 (11.5)	5 (5.3)	1 (5.0)
Urinary tract	6 (9.8)	6 (6.3)	2 (10.0)
Breast	4 (6.6)	2 (2.1)	2 (10.0)
Liver	3 (4.9)	8 (8.4)	2 (10.0)
Stomach	4 (6.6)	5 (5.3)	1 (5.0)
Other	9 (14.7)	27 (28.4)	3 (15.0)
Unknown	2 (3.3)	4 (4.2)	0 (0.0)
Not-applicable	3 (4.9)	11 (11.6)	2 (10.0)
Stage			
Local/loco-regional disease	16 (26.2)	20 (21.1)	7 (35.0)
Metastasic	42 (68.9)	64 (67.4)	11 (55.0)
Not-applicable	3 (4.9)	11 (11.6)	2 (10.0)
Preceding location			
Home	24 (39.3)	25 (26.3)	2 (10.0)
Hospital	37 (60.7)	70 (73.7)	18 (90.0)
Death during hospitalisation	28 (45.9)	87 (91.6)	15 (75.0)

^a^Higher scores indicate higher cognitive impairment.

^b^Higher scores indicate better performance status.

### Frequency of insomnia and other aspects in relation with insomnia

Results on the frequency of insomnia and other aspects in relation with insomnia are displayed in Table 2. Thirty-eight of the included patients (62.3%) answered “yes” to the insomnia single question, 13 patients (21.3%) answered “occasionally” and 10 patients (16.4%) answered “no”. Twenty-nine patients (47.5%) showed moderate to severe insomnia according to the SDS. The sleep item of the SDS that scored the highest was “waking up during the night”. Patients answering “yes” to the insomnia single question displayed significantly more frequently moderate to severe insomnia according to SDS in comparison to patients answering “occasionally” or “no” (78.9% vs 34.8%; p = 0.001).

**Table 2:** Frequency of insomnia and other aspects in relation with insomnia (n = 61)

**Frequency of insomnia**	
**Single question**	
Have you been suffering from insomnia since your admission?	**n (%)**
No	10 (16.4)
Occasionally	13 (21.3)
Yes	38 (62.3)
**Sleep disturbance scale**	
During the time you have been hospitalised have you suffered from…?	**n (%)**
Difficulty in falling asleep^a^ (mean ± SD)	4.5 ± 2.67
<5	28 (45.9)
≥5	33 (54.1)
Waking up during the night^a^ (mean ± SD)	4.9 ± 2.31
<5	23 (37.7)
≥5	38 (62.3)
Waking up too early in the morning^a^ (mean ± SD)	4.0 ± 2.65
<5	30 (49.2)
≥5	31 (50.8)
**Overall sleep disturbance scale score (range 0-10)**^**b **^**(mean ± SD)**	4.5 ± 1.96
<5	32 (52.5)
≥5	29 (47.5)
**Prevalence of additional aspects related to insomnia**	**n (%)**
Daytime sleepiness	45 (73.8)
Feeling of non-restorative sleep	32 (52.5)

^a^Likert 0-10.

^b^Overall Sleep Disturbance Scale score is calculated as the average of the rated scores of the three sleep items, a score ≥ 5 indicating moderate to severe insomnia.

Twenty-one patients (34.4%) reported < 5 hours sleep per night. Insomnia had often started together with the current disease (44.3%), and less frequently since admission to the PCU (11.5%) (results not shown in the table). Seventy percent of the patients were moderately worried about insomnia (information not shown in the table). Feeling of non-restorative sleep and daytime sleepiness were frequent (52.5% and 73.8% of the participants respectively).

### Frequency of emotional distress and other potentially associated factors

Results on the frequency of emotional distress and other factors potentially associated with insomnia are displayed in Table 3. The mean HADS score was 21.6 ± 7.66. Thirty-nine participants (63.9%) showed clinically significant emotional distress, 38 (62.3%) depressed mood and 33 (54.1%) anxiety. Nocturnal rumination and feelings of fear or loneliness were frequent. Pain was the most frequently claimed physical symptom interfering with sleep quality, followed by dyspnoea and cough. Most patients reported at least one environmental factor interfering with the quality of their sleep.

**Table 3:** Frequency of factors potentially associated with insomnia (n = 61)

	**Yes (n(%))**
**Psychological factors**	
HADS score (range 0-42)^a^ (mean ± SD)	21.6 ± 7.66
Clinically significant emotional distress^b^	39 (63.9)
Depressed mood^c^	38 (62.3)
Anxiety^d^	33 (54.1)
Nightmares	16 (26.2)
Rumination	48 (78.7)
Feelings of fear/loneliness	30 (49.2)
Clear knowledge diagnosis	39 (63.9)
Clear knowledge prognosis	12 (19.7)
**Physical symptoms claimed to be interfering with sleep quality**	
Pain	42 (68.9)
Dyspnoea	22 (36.1)
Cough	18 (29.5)
Nausea/vomiting	15 (24.6)
Sweating	13 (21.3)
Nycturia	11 (18.0)
Functional incontinence	8 (13.1)
Itch	7 (11.5)
Restless-legs	5 (8.2)
Drug/tobacco abstinence	5 (8.2)
Fever	1 (1.6)
**Environmental factors**	
Reported environmental factor influencing sleep quality	47 (77.0)
Individual room	28 (29.5)
Sleep habits not accomplished	19 (31.1)
Accompanied by a next of kin during night	15 (24.6)

^a^Hospital Anxiety and Depression Scale, higher scores indicate higher emotional distress.

^b^HADS score ≥ 19.

^c^HADD (subscale depression of HADS) ≥ 11.

^d^HADA (subscale anxiety of HADS) ≥ 11.

### Patients’ perspective on the group of factors that interfered most

When asked which group of factors interfered most with sleep quality, most patients (68.9%) mentioned the physical group; the environmental and psychological factors were mentioned less (11.5% and 9.8% respectively).

### Factors associated with insomnia

Table 4 shows the results of the univariate analysis for associations between the studied variables and insomnia or moderate to severe insomnia according to the single question and the SDS respectively. A higher HADS score and anxiety, but not clinically significant emotional distress or depression, were significantly associated with answering “yes” to the single insomnia question. Neither a higher HADS score, nor clinically significant emotional distress, anxiety or depressed mood were associated with moderate to severe insomnia measured by the SDS.

**Table 4:** Relationship between studied variables and insomnia according to the single question and the SDS (n = 61)

			**Insomnia (single question)**			**Moderate-severe insomnia (SDS)**		
			**No or occasionally (n (%))**	**Yes (n (%))**	**p-value**	**No (n (%))**	**Yes (n (%))**	**p-value**
**Socio-demographic/clinical characteristics**								
	**Mean age ± SD, years**		74.3 ± 9.73	69.8 ± 11.49	**0.072**	73.2 ± 9.95	69.6 ± 11.96	**0.289**
	**Sex**				**0.33**			**0.102**
		Women	12 (44.4)	15 (55.6)		11 (40.7)	16 (59.3)	
		Men	11 (32.4)	23 (67.6)		21 (61.8)	13 (38.2)	
	**Karnofsky index**				**0.003**^a^			**0.048**
		> 50	1 (6.3)	15 (93.8)		27 (60.0)	18 (40.0)	
		≤ 50	22 (48.9)	23 (51.1)		5 (31.3)	11 (68.8)	
	**Procedence**				**0.11**			**0.060**
		Home	12 (50.0)	12 (50.0)		9 (37.5)	15 (62.5)	
		Acute care	11 (29.7)	26 (70.3)		23 (62.2)	14 (37.8)	
	**Death during hospitalisation**				**0.768**			**0.088**
		No	13 (39.4)	20 (60.6)		18 (64.3)	10 (35.7)	
		Yes	10 (35.7)	18 (64.3)		14 (42.4)	19 (57.6)	
	**Cancer site**^b^				**0.056**			**0.534**
		Lung	2 (15.4)	11 (84.6)		8 (61.5)	5 (38.5)	
		Other	21 (46.6)	24 (53.3)		22 (48.9)	23 (51.1)	
		Non-applicable	0 (0.0)	3 (100.0)		2 (66.7)	1 (33.3)	
	**Stage**^b^				**0.239**			**0.888**
		Local or loco-regional disease	9 (56.3)	8 (43.8)		9 (56.2)	7 (43.5)	
		Metastasic	14 (33.3)	28 (66.7)		21 (50.0)	21 (50.0)	
		Non-applicable	0 (0.0)	3 (100.0)		2 (66.7)	1 (33.3)	
**Psychological factors**								
	**HADS** (range 0-42) (mean ± SD)		18.2 ± 7.64	23.7 ± 6.98	**0.012**	20.3 ± 7.79	23.1 ± 7.44	**0.158**
	**HADS ≥ 19**^c^				**0.137**			**0.806**
		No	11 (50.0)	11 (50.0)		12 (54.5)	10 (45.5)	
		Yes	12 (30.8)	27 (69.2)		20 (51.3)	19 (48.7)	
	**HADD ≥ 11**^d^				**0.204**			**0.306**
		No	11 (47.8)	12 (52.2)		14 (60.9)	9 (39.1)	
		Yes	12 (31.6)	26 (68.4)		18 (47.4)	20 (52.6)	
	**HADA ≥ 11**^e^				**0.019**			**0.234**
		No	15 (53.6)	13 (46.4)		17 (60.7)	11 (39.3)	
		Yes	8 (24.2)	25 (75.7)		15 (45.4)	18 (54.5)	
	**Nightmares**				**0.535**			**0.163**
		No	18 (40.0)	27 (60.0)		26 (57.8)	19 (42.2)	
		Yes	5 (31.3)	11 (68.8)		6 (37.5)	10 (62.5)	
	**Rumination**				**0.012**^a^			**0.009**^a^
		No	9 (69.2)	4 (30.8)		11 (86.4)	2 (15.4)	
		Yes	14 (29.2)	34 (70.8)		21 (43.8)	27 (56.3)	
	**Feelings of fear/loneliness**				**0.222**			**0.055**
		No	14 (45.2)	17 (54.8)		20 (64.5)	11 (35.5)	
		Yes	9 (30.0)	21 (70.0)		12 (40.0)	18 (60.0)	
	**Clear knowledge of diagnosis**				**0.042**			**0.065**
		No	12 (54.5)	10 (45.5)		17 (43.6)	22 (56.4)	
		Yes	11 (28.2)	28 (71.8)		15 (68.2)	7 (31.8)	
	**Clear knowledge of prognosis**				**0.751**			**0.404**
		No	18 (36.7)	31 (63.3)		5 (41.7)	7 (58.3)	
		Yes	5 (41.7)	7 (58.3)		27 (55.1)	22 (44.9)	
**Physical factors**^f^								
	**Pain**				**0.633**			**0.986**
		No	8 (42.1)	11 (57.9)		10 (52.6)	9 (47.4)	
		Yes	15 (35.7)	27 (64.3)		22 (52.4)	20 (47.6)	
	**Dyspnoea**				**0.018**^a^			**0.436**
		No	19 (48.7)	20 (51.3)		19 (58.7)	20 (51.3)	
		Yes	4 (18.2)	18 (81.8)		13 (59.1)	9 (40.9)	
	**Cough**				**0.649**			**0.381**
		No	17 (39.5)	26 (60.5)		21 (48.8)	22 (51.2)	
		Yes	6 (33.3)	12 (66.7)		11 (61.1)	7 (38.9)	
	**Nausea/vomiting**				**0.150**			**0.501**
		No	15 (32.6)	31 (67.4)		23 (50.0)	23 (50.0)	
		Yes	8 (53.3)	7 (46.7)		9 (60.0)	6 (40.0)	
	**Sweating**				**0.530**			**0.910**
		No	17 (35.4)	31 (64.6)		25 (52.1)	23 (47.9)	
		Yes	6 (46.2)	7 (53.8)		7 (53.8)	6 (46.2)	
	**Nycturia**				**1.000**			**0.412**
		No	19 (38.0)	31 (62.0)		25 (50.0)	25 (50.0)	
		Yes	4 (36.0)	7 (63.6)		7 (63.6)	4 (36.4)	
**Environmental factors**								
	**Environmental disturbances**				**0.650**			**0.026**
		No	6 (42.9)	8 (57.1)		11 (78.6)	3 (21.4)	
		Yes	17 (36.2)	30 (63.8)		21 (44.7)	26 (55.3)	
	**Individual room**				**0.301**			**0.754**
		No	18 (41.9)	25 (58.1)		22 (51.2)	21 (48.8)	
		Yes	5 (27.8)	13 (72.2)		10 (55.6)	8 (44.4)	
	**Accompanied by next of kin during night**				**0.310**			**0.204**
		No	19 (41.3)	27 (58.7)		22 (47.8)	24 (52.2)	
		Yes	4 (26.7)	11 (73.3)		10 (66.7)	5 (33.3)	
	**Sleeping habits not accomplished**				**0.217**			**0.276**
		No	18 (42.9)	24 (57.1)		24 (57.1)	18 (42.9)	
		Yes	5 (26.3)	14 (73.7)		8 (42.1)	11 (57.9)	
**Additional aspects related to insomnia**								
	**Daytime sleepiness**				**0.237**			**0.010**^a^
		No	8 (50.0)	8 (50.0)		4 (25.0)	12 (75.0)	
		Yes	15 (33.3)	30 (66.7)		28 (62.2)	17 (37.8)	
	**Feeling of non-restorative sleep**				**0.007**			**0.913**
		No	16 (55.2)	13 (44.8)		15 (51.7)	14 (48.3)	
		Yes	7 (21.9)	25 (78.1)		17 (53.1)	15 (46.9)	

^a^Statistical significance persisted after multivariate analysis.

^b^Statistical analysis excluded the 3rd group (non-applicable).

HADS: Hospital Anxiety and Depression Scale (higher scores indicate higher emotional distress).

^c^Indicating clinically significant emotional distress.

HADD: subscale Depression of HADS.

^d^Indicating depressed mood.

HADA: subscale Anxiety of HADS.

^e^Indicating anxiety.

^f^Only those reported by > 8 patients presented.

In the multivariate regression analysis, insomnia according to the single question remained associated with dyspnoea (OR 7.2 [1.65-31.27] p = 0.009), nocturnal rumination (OR 5.5 [1.05-28.49] p = 0.04) and higher performance status (OR 14.3 [1.62-125.43] p = 0.017). Moderate to severe insomnia according to the SDS was associated with nocturnal rumination (OR 5.6 [1.1-29.1]; p = 0.041), and inversely associated with daytime sleepiness (OR 0.25 [0.07-0.9]; p = 0.043).

## Discussion

The frequency of insomnia among the patients admitted to the Palliative Care Unit (PCU) was considerably high both when measured with the single question (62.3%) and with the Sleep Disturbance Scale (SDS) (47.5% moderate to severe insomnia). Results of the two instruments correlated significantly with each other. These results are comparable to those on insomnia prevalence existing in the literature. Studies using the Pittsburg Sleep Quality Index (PSQI) found prevalences of 73% to 96% of “poor sleepers” using different cut-off scores [5,11]. Other studies showed prevalences of 72% and 45% using a constructed questionnaire and one item of the major depressive episode module of the Structured Clinical Interview for DSM-IIIR (SCID), respectively [4,25]. A prevalence of 70% was found in another study by asking the single question “Do you have trouble sleeping at night?” [12].

The single question “Have you been suffering from insomnia since your admission?” might be a sensitive method for detecting insomnia. However, the use of a single question has been considered as insufficient for the evaluation of insomnia in cancer patients, as it may have poor sensitivity and specificity for detecting insomnia [32]. In the present study, patients with higher HADS score, more anxiety, clear knowledge of the diagnosis, better performance status, nocturnal rumination, “dyspnoea” as a symptom interfering with sleep quality, and a feeling of non-restorative sleep answered more often “yes” to this single question in the univariate analysis.

The SDS assesses the presence of moderate to severe insomnia, is easy and quick to administrate, refers to a short recent period, and gives information about the three sleep items: falling asleep, waking up during the night and early waking. The use of a Likert scoring system allows participants to respond in a degree of agreement accommodating neutral or undecided feelings, and without requiring them to provide a simple and concrete yes or no answer [33]. Therefore, the assessment of insomnia of patients during admission in a PCU using SDS may have some advantages over the insomnia single question and other tools which take longer to respond to and refer to a longer preceding period of time. In the present study, patients with better performance status, nocturnal rumination, patients referring environmental disturbances and daytime sleepiness showed more moderate to severe insomnia in the univariate analysis.

The most affected sleep item was sleep maintenance, followed by difficulties in falling asleep and early waking, coinciding with data from other studies on patients with cancer [9,14].

Clinically significant emotional distress was frequent in this study. A higher HADS score and the presence of anxiety were associated with answering “yes” to the insomnia single question in the univariate analysis, but this association was not found in the multivariate analysis, and no association was found with clinically significant emotional distress. Moderate to severe insomnia measured with the SDS was not associated with clinically significant emotional distress, higher HADS score, anxiety or depression. These results contrast with those in previous studies reporting on a correlation between insomnia and anxiety and/or depression measured with an analogue visual scale as part of the Edmonton Symptom Assessment System [34] in palliative patients [13,16], or with a recent study reporting on a correlation between insomnia severity and depression measured using a non-validated tool [35]. Another recent study found a significant correlation between emotional distress measured with the HADS and insomnia measured with Athens Sleep Insomnia Scale [36] among patients with lung cancer [37].

Nevertheless, in the present study the psychological symptom nocturnal rumination did show a significant association with insomnia assessed with both instruments. In the unwanted mental activity at bedtime, rumination is a dysfunctional control strategy used by poor sleepers, which maintains cognitive and affective arousal instead of helping them to relax [3,38]. The association between rumination and insomnia is consistent throughout the literature [3,12,14,31,38,39]. Rumination has been associated with an increased and prolonged emotional distress (i.e. depression and anxiety) and with physical health problems in palliative care patients [40] and in the general population [38,41,42]. However, some recent studies have suggested that rumination may contribute to clinical insomnia, irrespective of worry and depressed mood states [39,43,44].

Different ways of assessing rumination have been proposed in the literature [31,41,43,45], but only few have been used in palliative care patients [30,40]. In the present study, rumination was evaluated by means of a simple question based on a definition obtained from the literature [30,31]. Several methods for managing rumination have been also developed, and it has been proposed that its treatment may be imperative for a successful management of insomnia and depression [31,45]. Strategies aimed at improving rumination could be included into the cognitive behavioral therapy of insomnia [31,45].

Despite the association and possible role of rumination in connection with insomnia, patients in this study claimed physical factors to be the type of symptoms that interfered most with their sleep quality, coinciding with results reported in other studies [12]. This result suggests that patients may tend to underreport psychological symptoms such as rumination, but also highlights the importance of careful evaluation of physical symptoms. Among the physical factors, pain was the symptom that patients mentioned most often as interfering with their sleep quality, suggesting that pain should be especially taken into consideration when assessing and managing insomnia, as reported in other studies [11,14]. Nevertheless, pain was not found associated with insomnia in the multivariate regression analysis in the present study.

Dyspnoea showed a significant association with insomnia measured with the single question in the multivariate regression analysis. Dyspnoea has been also reported in the literature as being the cause of sleep disturbance [12,46]. However, this association has not been confirmed in other studies [4]. Also a higher performance status (higher Karnofsky index) has shown association with insomnia measured with the single question in the multivariate regression analysis of this study. Unlike this study, other studies found that a lower performance status might be associated with insomnia [4,11]; however, these results were not confirmed in the multivariate analyses of these studies. Results regarding the Karnofsky index in the present study should be interpreted cautiously, as the range of performance status included was limited by the inclusion criteria.

Patients with daytime sleepiness in this study showed moderate to severe insomnia less often according to the SDS, in comparison to those patients without daytime sleepiness (37.8% vs 75.0%), suggesting that patients with moderate to severe insomnia might be more often awake in PCUs during the daytime. In a secondary analysis, daytime sleepiness was associated with a higher intake of psychotropic medication; it could be hypothesized that sleepiness due to psychotropic medication might also be happening at night. Literature on the relationship between daytime sleepiness and insomnia in palliative care is scarce. Although there is some controversy, our result is comparable with other studies [46,47]. Nevertheless, excessive daytime sleepiness in the general adult population seems more likely to be attributed to short sleep duration [48], but the insufficient sleep can be due to insomnia or to other factors. Pharmacological treatment and biological aspects associated with advanced cancer, such as cytokines influencing the sleep-wake cycle [49], could influence the presence of daytime sleepiness. Further studies are needed in order to understand the relationship between daytime sleepiness and insomnia.

To the best of our knowledge, environmental factors have rarely been considered in the literature in relation to insomnia. Despite not being significantly associated with insomnia in the multivariate analysis, results of this study show that patients do care about environmental factors with regard to their sleep. Although PCUs are generally quieter than other conventional hospital wards, this result suggests that special care should be taken to ensure that these units are free of unnecessary noise, light or other interruptions during the night-time. As suggested by the hyperarousal theory, patients with insomnia might find themselves in a cycle of increased cognitive and physiological sustained sensory processing of environmental stimuli [50,51].

In the present study no association between insomnia and other sociodemographic (e.g. gender, age) or clinical characteristics (e.g. diagnosis and stage of the disease, medication use) was found.

This study has limitations. The low sample size does not allow us to speak about prevalence of insomnia and limits the interpretation of the results of the multivariate analysis. This study should be seen as exploratory. Replication of the results in further studies with larger sample sizes is required. The SDS was translated into the Catalan language, but no backward translation was performed, as recommended by methodological literature [52]. However, the items were not complex in terms of language and no important cultural differences were expected after translation. Furthermore, no formal validation of the scale was carried out.

The day during admission on which interviews took place varied, with some taking place after one week of admission. Only 36% of the consecutively admitted patients were included in the study. About 10% of the admitted patients were not assessed for inclusion because they were discharged or died before, but their sociodemographic characteristics did not differ from the other patients admitted. More than half of the admitted patients were excluded because they were too confused or had a too bad performance status, similar to that experienced in other studies [4]. Results of the present study may not be representative of all patients admitted, but of those with better cognitive and performance status.

## Conclusions

In conclusion, this study suggests a high frequency of insomnia among palliative care patients during their admission in a PCU, both when measured using a short single question and the Sleep Disturbance Scale. Furthermore, a high frequency of clinically significant emotional distress (including anxiety and depression) was shown. Pain, dyspnoea and environmental factors were often mentioned by patients as interfering with their sleep quality, and patients mentioned physical factors as the type of factor interfering most. While clinically significant emotional distress was not associated with insomnia in this study, nocturnal rumination was associated with insomnia when measured with both tools and this association remained in the multivariate analysis. The assessment of nocturnal rumination might be of particular interest in the management of insomnia. These results should be confirmed in further prospective studies with a larger sample size.

## Abbreviations

CRF: Case Report Form; DSM-IIIR: Diagnostic and Statistical Manual of Mental Disorders-IIIR; HADS: Hospital Anxiety and Depression Scale; HADA: subscale Anxiety of HADS; HADD: subscale Depression of HADS; PCU: Palliative Care Unit; SCID: Structured Clinical Interview for DSM-IIIR; SDS: Sleep Disturbance Scale.

## Competing interests

The authors declare that there is no conflict of interest.

This research received no specific grant from any funding agency in the public, commercial, or not-for-profit sectors.

## Authors’ contributions

ARG, ARR, JP and CF developed the research question and conceived the study design. ARG and MS collected and managed the data. ARG, JP, ARR and SM performed the data analysis. ARG drafted the manuscript, supported by ARR, JP, CF and RM. All authors critically read and approved the final manuscript.

## Pre-publication history

The pre-publication history for this paper can be accessed here:

http://www.biomedcentral.com/1472-684X/13/40/prepub
